# Overcoming Challenges with Biochemical Studies of Selenocysteine and Selenoproteins

**DOI:** 10.3390/ijms251810101

**Published:** 2024-09-20

**Authors:** Antavius Cain, Natalie Krahn

**Affiliations:** Department of Biochemistry and Molecular Biology, University of Georgia, Athens, GA 30602, USA; ac14754@uga.edu

**Keywords:** selenocysteine, biochemistry, selenoprotein, selenium, redox, translation

## Abstract

Selenocysteine (Sec) is an essential amino acid that distinguishes itself from cysteine by a selenium atom in place of a sulfur atom. This single change imparts distinct chemical properties to Sec which are crucial for selenoprotein (Sec-containing protein) function. These properties include a lower p*K*_a_, enhanced nucleophilicity, and reversible oxidation. However, studying Sec incorporation in proteins is a complex process. While we find Sec in all domains of life, each domain has distinct translation mechanisms. These mechanisms are unique to canonical translation and are composed of Sec-specific enzymes and an mRNA hairpin to drive recoding of the UGA stop codon with Sec. In this review, we highlight the obstacles that arise when investigating Sec insertion, and the role that Sec has in proteins. We discuss the strategic methods implemented in this field to address these challenges. Though the Sec translation system is complex, a remarkable amount of information has been obtained and specialized tools have been developed. Continued studies in this area will provide a deeper understanding on the role of Sec in the context of proteins, and the necessity that we have for maintaining this complex translation machinery to make selenoproteins.

## 1. Introduction

Selenocysteine (Sec) is a pivotal amino acid ubiquitous across all forms of life. It is uniquely encoded by the UGA stop codon following a strategic mechanism. The chemical structure of Sec is similar to the structure of cysteine (Cys), but it is distinguished through the substitution of the sulfur (S) atom for a selenium (Se) atom. This single change is the underlying reason for its distinct chemistry [1]. This substitution yields a lower p*K*_a_ value for Sec (5.2 vs. 8.3), which at physiological pH makes it more nucleophilic (carrying a negative charge) than Cys [2,3]. Sec is also readily oxidized to form a diselenide bond (Se–Se), that can be harnessed in redox reactions and enhance protein stability. Finally, the larger atomic radius of Se, compared to S, makes it more electrophilic [4]. Combined, these features increase the chemical reactivity of Sec and, through integrating it into proteins (selenoproteins), imparts these proteins with enhanced biological functions [5].

**Figure 1:**
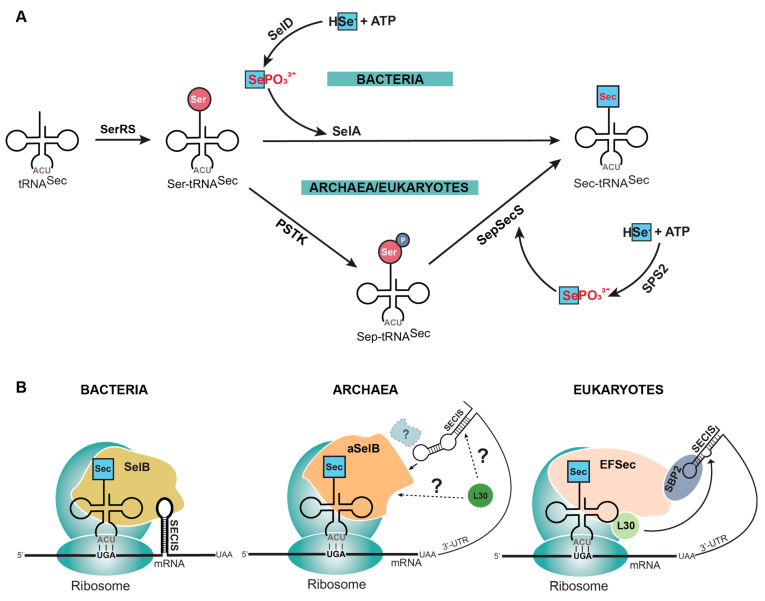
Natural insertion of selenocysteine (Sec) into proteins. (**A**) Sec is synthesized on its tRNA (tRNA^Sec^) because of the labile nature of the free amino acid (red letters). This requires tRNA^Sec^ to first be aminoacylated with serine (Ser) by seryl-tRNA synthetase (SerRS). Ser is then converted to Sec in a single step in bacteria by selenocysteine synthase (SelA) or two steps in archaea and eukaryotes by *O*-phosphoseryl-tRNA^Sec^ kinase (PSTK) and *O*-phosphoserine tRNA^Sec^: selenocysteine synthase (SepSecS). Selenophosphate (SePO_3_^3−^) is the donor molecule required for converting Ser to Sec, but this is also labile (red letters) and therefore is generated by a separate enzyme (SelD or SPS2) from elemental selenium (HSe^−^). (**B**) The process of bringing Sec-tRNA^Sec^ to the ribosome for translation requires multiple factors that differ depending on the domain of life. Briefly, Sec-tRNA^Sec^ is recognized by a specialized elongation factor (SelB, aSelB or EFSec) and is directed to a UGA codon by an mRNA hairpin (SECIS element) required in the translated (bacteria) or untranslated region (archaea and eukaryotes) of the mRNA.

The process of canonical protein synthesis, involving translation of an mRNA sequence into protein, is well-documented [6,7]. However, selenoprotein translation entails additional enzymes and regulatory elements that are absent in the canonical translation machinery. First, Sec does not have its own aminoacyl-tRNA synthetase (aaRS), and instead utilizes endogenous seryl-tRNA synthetase (SerRS) to aminoacylate its tRNA (tRNA^Sec^) with serine (Ser). Second, Ser has to be converted to Sec, which occurs in a single step in bacteria catalyzed by selenocysteine synthase (SelA), or in two steps in eukaryotes and archaea catalyzed by *O*-phosphoseryl-tRNA^Sec^ kinase (PSTK) and *O*-phosphoserine tRNA^Sec^: selenocysteine synthase (SepSecS) (Figure 1A) [8]. Third, to distinguish which UGA codon is to be recoded with Sec, the mRNA sequence contains a Sec insertion sequence (SECIS) element. This element is found directly downstream of the UGA codon in the translated region of bacterial mRNAs, and in eukaryotes in the 3′ untranslated region (UTR) of selenoprotein mRNAs [9]. Most archaeal selenoproteins also have the SECIS in the 3′ UTR, though there is an observed occurrence of the SECIS being located in the 5′ UTR [10]. Finally, the elongation factor (SelB in bacteria, aSelB in archaea, or EFSec in eukaryotes) emerges as a linchpin in selenoprotein synthesis. In bacteria, SelB directly interacts with Sec-tRNA^Sec^ and the SECIS element for recoding of the UGA codon. In eukaryotes, EFSec interacts with Sec-tRNA^Sec^ and the SECIS-binding protein (SBP2) which, after forming a complex, interacts with the SECIS element to facilitate Sec insertion (Figure 1B) [11,12]. The coordinated interplay of these elements is fundamental to the intricate machinery governing selenoprotein synthesis.

## 2. The Instability of Selenocysteine

The amino acid Sec is unique among the 20 canonical amino acids, in that it is not commercially available. This is because of its propensity to oxidize in air. As a result, it is more common to find the oxidized derivative, selenocystine, which has a Se–Se bond, than the reduced Sec. For the same reason, Sec is not readily found in a cellular amino acid pool. Nature has instead evolved a strategic aminoacylation path to handle these oxidation properties of Sec, which takes advantage of the structural similarities between Ser and Sec. Just as there is a single difference in Cys compared to Sec, the same is true for Ser, containing oxygen instead of Se. Therefore, tRNA^Sec^ is first aminoacylated with Ser via SerRS, and then Ser is converted to Sec. This occurs in a single step in bacteria using SelA, or in two separate steps in archaea and eukaryotes using PSTK to generate phosphoserine (Sep) and then SepSecS to convert Sep to Sec (Figure 1A).

Both SelA [13] and SepSecS [14,15] are fold-type-I pyridoxal 5′-phosphate (PLP)–dependent enzymes that use selenophosphate as a selenium donor to convert Ser or Sep to Sec, respectively. However, selenophosphate is also labile at neutral pH and therefore not found in the cellular amino acid pool for protein synthesis [16]. The incorporation of inorganic selenite into selenophosphate, essential for selenoprotein biosynthesis, involves several steps, many of which remain unresolved. Selenite is first reduced to selenide (H_2_Se) by cellular redox systems, like thioredoxin or glutathione. Selenide then serves as a substrate for selenophosphate synthetase (SPS2 in mammals or SelD in bacteria), which catalyzes the formation of selenophosphate by transferring a phosphate group from ATP to selenide in a reaction that produces AMP and SePO^3−^. This process requires ATP binding and hydrolysis, facilitated by Mg^2+^ ions, and is thought to involve a flexible N-terminal loop containing a key Cys or Sec. NifS-like proteins may also assist by converting Sec to H_2_Se; the exact nature of selenium delivery, and certain mechanistic details of ATP hydrolysis and product release remain unclear [17] (Figure 1A). This necessity for the tRNA biosynthesis of Sec is unlike canonical tRNA aminoacylation, which occurs in a single step using a dedicated aaRS and its respective amino acid. As a result, the study of selenoprotein synthesis has required unique strategies and tools to understand the synthesis mechanism, and to establish expression systems for producing recombinant selenoproteins.

## 3. Biochemical Studies

The mechanism of selenoprotein synthesis differs in each domain of life and has subsequently been studied to different degrees. Arguably, the simplest and most well-characterized mechanism is in bacteria [18]. Archaea is the least characterized with the question still proposed if all proteins involved in the process have been identified [10]. Eukaryotic Sec insertion is still heavily studied with new structural data recently released that improves our mechanistic understanding [12]. In addition to understanding the natural translation mechanism, interest in the study of selenoproteins in vitro is also growing. As described above, Sec is labile and not able to be added to experimental setups to study selenoprotein translation. As a result, the standard protocol used for canonical protein translation, in which amino acids can be commercially purchased and added into a reaction, is not a viable option. Instead, novel strategies have been implemented for studying selenoprotein formation. In some cases, Sec-tRNA^Sec^ was prepared for the experiment, but often Ser-tRNA^Sec^ was used instead; in rare cases, unaminoacylated tRNA^Sec^ is used. Below we provide a critical review on the choice of substrate for the types of experiments and the considerations needed when analyzing the resulting data.

### 3.1. Studies Directly with Selenocysteine

Studying the biosynthesis of Sec and the mechanism by which it is inserted into a polypeptide chain boasts the importance of using the correct amino acid (Sec) in an experiment. This requires the formation of Sec-tRNA^Sec^, or if investigating the mammalian or archaeal system, could also involve the formation of Sep-tRNA^Sec^.

#### 3.1.1. Aminoacylation Studies

The secondary structure of tRNA is highly conserved; however, there are distinguishing features of each tRNA that promote interaction with their cognate aaRS for aminoacylation with the correct amino acid [19]. The same is true when it comes to tRNA^Sec^; however, there is not just a single enzyme that is required for aminoacylation, but rather two (in bacteria) or three (in eukaryotes and archaea) that are essential for full selenocysteinylation. The initial aminoacylation step, serylation of tRNA^Sec^, can and has been studied with standard in vitro methods [20]. However, to investigate the subsequent step(s) of phosphorylation and selenocysteinylation, the starting reagent needs to be self-prepared. In bacteria, this requires initial aminoacylation of tRNA^Sec^ with Ser to study the conversion of Ser to Sec. In eukaryotes and archaea, Ser to Sec conversion occurs in two separate steps (phosphorylation then selenocysteinylation), so these steps need to be investigated individually with separate starting materials.

**Figure 2:**
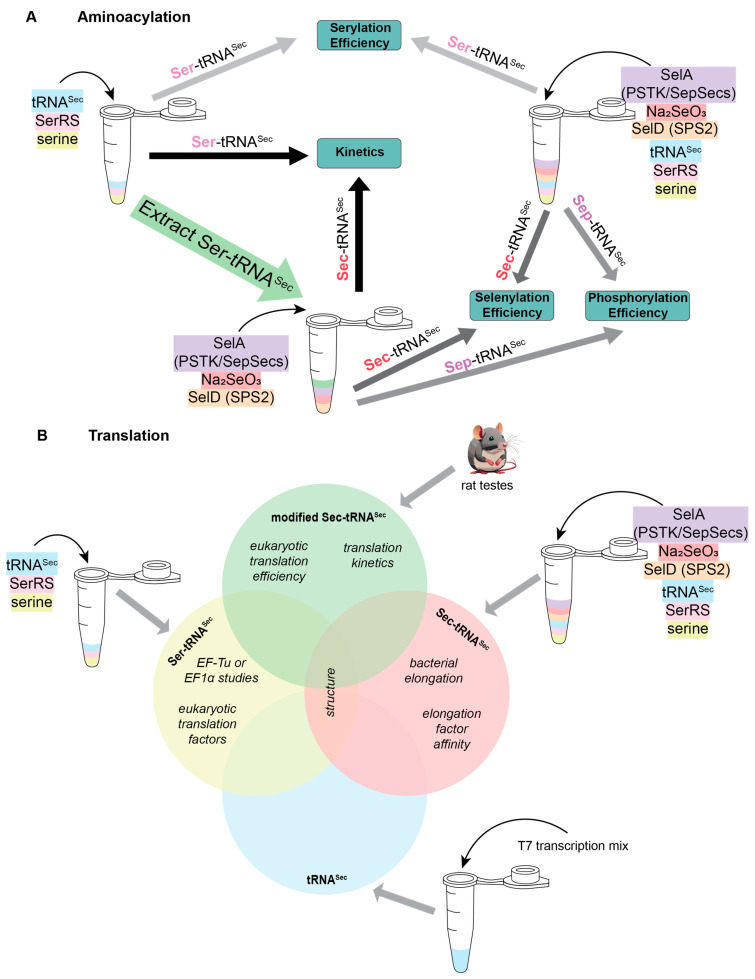
Schematic of experimental requirements required to study various stages of (**A**) aminoacylation and (**B**) translation for selenoproteins. Molecules are color-coordinated to follow when they are needed.

As with many reactions, aminoacylation of tRNA^Sec^ with Ser is not 100% efficient. Therefore, when studying phosphorylation by PSTK or selenocysteinylation by SelA, it is important to decide whether the separation of unacylated tRNA^Sec^ from Ser-tRNA^Sec^ is necessary to answer the proposed experimental question. The standard practice for aminoacylation reactions is to mix tRNA, aaRS, and amino acid (of which the tRNA or amino acid is radioactive) in the presence of ATP and MgCl_2._ After the reaction has been completed, the tRNA is digested with P1 nuclease to release the amino acid attached to the tRNA. Quenched reactions are then spotted onto a chromatography plate and the amino acids are resolved from AMP. The solvent used for this chromatographic step causes Ser, Sep, and Sec to travel at different rates, distinguishing the three amino acids from one another. This property of chromatography allows reaction efficiencies to be calculated by the intensity ratio of the corresponding amino acids. It is a useful strategy that prevented the need for the initial serylation step to be purified before investigating identity elements of phosphorylation and selenocysteinylation for human tRNA^Sec^ [21,22,23]. On the other hand, kinetic data cannot be determined in this way, because the exact concentration of starting material (Ser-tRNA^Sec^ or Sep-tRNA^Sec^) in the reaction is not known. In situations such as these, purification of deacylated tRNA^Sec^ from Ser-tRNA^Sec^ (or Ser-tRNA^Sec^ from Sep-tRNA^Sec^) must be performed (Figure 2A).

Moreover, in these reactions there is a requirement of a selenophosphate pool for conversion of the attached amino acid to Sec. As discussed above, selenophosphate, like Sec, is not stable in solution. Therefore, in vitro reactions require an additional enzyme to produce this substrate. In bacteria, the enzyme to produce selenophosphate is SelD, while in eukaryotes and archaea it is SEPHS2 (also referred to as SPS2) [24]. With this information, studying an intermediary step in the Sec biosynthesis pathway of bacteria requires the pre-formation of Ser-tRNA^Sec^ plus the addition of SelD, SelA, and sodium selenite. A similar strategy was used for understanding the mechanism of SepSecS, but with the pre-formation of Sep-tRNA^Sec^ by SerRS and PSTK before adding in SepSecS, SelD, and sodium selenite (Figure 2A) [8].

#### 3.1.2. Translation Studies

After aminoacylation, Sec-tRNA^Sec^ is inserted into the ribosome through the Sec-specific elongation factor (SelB or EFSec). Canonical elongation factors rely on the presence of aminoacylated tRNA for binding and have increased affinity compared to deacylated tRNA [25]. The same is true for Sec-specific elongation factors; however, their affinity is further influenced by which amino acid is attached to tRNA^Sec^. SelB and EFSec preferentially bind Sec-tRNA^Sec^ over Ser-tRNA^Sec^, but recent studies suggest that EFSec may recognize Ser-tRNA^Sec^ with a higher affinity than SelB [12]. Detailed kinetic analysis of the EFSec interaction between Ser-tRNA^Sec^ compared to Sec-tRNA^Sec^ is not known. To determine this, prior preparation of aminoacylated tRNA^Sec^ is necessary. A one-pot reaction mix containing SerRS, PSTK, SepSecS, and SPS2 with tRNA^Sec^ facilitates full selenocysteinylation, while serylation requires only SerRS. Afterwards, the reaction is followed by chromatography to remove the deacylated tRNA, resulting in pure Sec-tRNA^Sec^ or Ser-tRNA^Sec^ for experimentation [26]. Following this strategy (but using SerRS, SelA, and SelD for bacterial selenocysteinylation), it was determined that SelB recognizes Sec-tRNA^Sec^ with 10^6^ times higher affinity than Ser-tRNA^Sec^. This means that, when studying elongation in bacteria, it is recommended to work with Sec-tRNA^Sec^ (Figure 2B). However, using Sec-tRNA^Sec^ adds its own complications because Sec is labile. It is presumed that Sec is stabilized when it is on tRNA^Sec^, but its stability has not been well-characterized. Some studies have been conducted anaerobically to prevent oxidation of the Sec [8,27,28]; nonetheless, there is no evidence that this is necessary [15,21]. Presumably, since selenoproteins are made in humans in an aerobic environment, Sec-tRNA^Sec^ is stable long enough to be incorporated into the polypeptide chain and therefore, working anaerobically, is not necessary. With regards to EFSec, detailed binding affinity studies have not been conducted, though the recent literature suggests that there is sufficient recognition of Ser-tRNA^Sec^ by EFSec to allow for both in vivo and in vitro studies [12].

In addition to understanding the binding affinity and kinetics of the Sec-specific elongation factors with tRNA^Sec^, there is considerable research to study the overall translation efficiency. To study this in vitro, Sec-tRNA^Sec^ must be available. We have already discussed how this can be performed using a one-pot reaction, in some situations it may be preferrable to work with native tRNA^Sec^. Native tRNA^Sec^ is modified, with different modification patterns in bacteria [29] compared to vertebrate tRNA^Sec^ [30]. There are, however, two modifications in the anticodon loop that are conserved between them: hypermodified 5-methylcarboxymethyl (mcm^5^)U34, which may be further methylated on the 2′O-position of the ribose (mcm^5^Um34); and N^6^-isopentenyl(i^6^)A37 [30]. The U34 and i^6^A37 modifications have been connected to an increase in selenoprotein expression due to increased UGA suppression [31,32,33,34,35]. Structural data on the ribosome shows that the i^6^A37 modification stacks on the anticodon–codon minihelix [36], while solution data shows that mcm^5^Um34 can position the anticodon in an optimal position for decoding [37].

The importance of these modifications for decoding UGA emphasizes the need for native tRNA^Sec^. Initial methods to achieve this relied on the purification of native tRNA^Sec^ from rat testes, a rich source of Sec-tRNA^Sec^ (Figure 2B) [38]. This process is labor intensive and requires three column purifications before it can be used for further experimentation. Because of the multiple steps, the resulting yield of Sec-tRNA^Sec^ is low, and is often the limiting factor in these reactions. Further understanding of Sec translation has allowed optimization of this process: specifically, the interaction of Sec-tRNA^Sec^ with EFSec. By tagging EFSec with a FLAG tag it facilitated affinity purification of Sec-tRNA^Sec^ from rat testes, increasing yields 35-fold [39]. This strategy had a two-fold success rate: (i) it drastically improved the amount of natively modified Sec-tRNA^Sec^ purified from rat testes; and (ii) it proved that EFSec/GTP/tRNA form an active ternary complex. With this complex, understanding the delivery of Sec-tRNA^Sec^ to the ribosome was made possible [39].

Modifications in *E. coli* tRNA^Sec^ have also been characterized as follows: dihydrouridine at position 20, ribothymidine at position 54, pseudouridine at position 55, and i^6^A37 [29]. However, the details of their role in translation are not as heavily studied [30]. Generally, i^6^A37 has been found to affect translation efficiency, especially with regards to the tRNA suppression of stop codons [40]. Therefore, accurate information for the study of translation kinetics should use tRNA^Sec^ extracted from a native host. With regards to the study of elongation factor affinity, it has been shown that binding SelB to unmodified Sec-tRNA^Sec^ was equivalent to native modified Sec-tRNA^Sec^ [26].

### 3.2. Studies without Selenocysteine as a Substrate

The challenges associated with the production of Sec-tRNA^Sec^ were avoided in some biochemical studies. These studies focus on understanding the mechanism of translation and cannot provide information on the kinetics of translation. When investigating the natural system for translation of eukaryotic selenoproteins, an in vitro reporter was used to determine the requirement of the SECIS element and SBP2 for selenoprotein synthesis [12]. Since EFSec is still capable of binding Ser-tRNA^Sec^, and both the SECIS element and SBP2 do not recognize the amino acid, then the preparation of Sec-tRNA^Sec^ could be avoided.

A similar strategy was used during the development of a SECIS-independent translation system that uses the natural elongation factor (described below) to recognize an engineered tRNA^Sec^ (allo-tRNA). Unlike SelB and EFSec, which have a higher affinity for Sec over Ser, the natural elongation factor does not. EF-Tu (the natural elongation factor in bacteria) distinguishes less between what amino acid is attached, but differentiates on the presence vs. absence of one [41]. Therefore, when studying the translation efficiency of allo-tRNA, Ser-allo-tRNA was used for single-molecule experiments, rather than preparing Sec-allo-tRNA [42].

Another situation in which the amino acid may not be critical is when gaining structural information. Preparing materials for crystallization requires high concentrations of material, that can often induce binding of tRNA to the elongation factor regardless of whether the amino acid is present. This is true for SelB binding to tRNA^Sec^ as the K_d_ for deacylated tRNA^Sec^ is roughly 500 nM, which can easily be overcome with crystallization concentrations [26]. Therefore, it is often seen that Ser [12], or even no amino acid [43] is used when making complex structures for determination of interaction sites in Sec translation.

## 4. Expression of Selenoproteins In Vivo

Inserting Sec naturally into proteins occurs at the UGA stop codon. However, to prevent insertion at all UGA codons, there is an additional requirement of a SECIS element that is recognized by proteins involved in elongation [12,44]. In bacteria, the SECIS element is found directly downstream of the UGA codon, in the translated region of the mRNA; while in archaea and eukaryotes, the SECIS element is typically found in the 3′-untranslated region of the mRNA (Figure 1B) [10]. Since the mechanism for Sec insertion in each domain of life differs, it restricts heterologous selenoprotein expression. Often eukaryotic or archaeal proteins are expressed in a bacterial host (such as *E. coli*) but, when it comes to selenoproteins, the selenoprotein gene cannot simply be transplanted into a plasmid for *E. coli* expression.

### 4.1. Installing a SECIS Element

#### 4.1.1. Bacteria

In bacteria, SelB is the only enzyme required for the elongation of Sec; it recognizes Sec-tRNA^Sec^ and the SECIS element for insertion of Sec at the UGA codon. For this to happen, all must be in close proximity to each other, restricting the SECIS element to be 11 nts from the UGA codon [45]. Bacterial selenoprotein mRNAs are designed for this with a SECIS element already encoded at the correct position in the mRNA. However, when trying to express a eukaryotic selenoprotein or to install Sec into a non-selenoprotein, the SECIS element would be either too far away to be useful for SelB recognition or non-existent, respectively.

Three strategies have been employed to tackle this predicament using the natural Sec machinery to install a SECIS element as follows: (i) into the translated region of the mRNA, (ii) into the untranslated region of the mRNA, or (iii) into the ribosomal RNA (rRNA). Detailed studies found that the entirety of the SECIS element is not critical for function, but rather can be minimized to a smaller size and still promote Sec insertion. This minimal requirement of the SECIS is the upper stem-loop structure (17 nts), which recruits and binds to SelB. Installing even a minimal SECIS element downstream of the UGA codon can mutate the resulting protein sequence. Regardless, this strategy has been shown to be possible with glutathione-*S*-transferase, without affecting its glutathione-binding capacity, marking it the first heterologous selenoprotein expression in *E. coli* using an internal Sec residue [45]. Methionine-*R*-sulfoxide reductase, a eukaryotic selenoprotein, was also actively expressed in *E. coli* using an internal SECIS element. In this case, instead of introducing 17 nts, the sequence downstream of the UGA was minimally mutated to produce a SECIS element [46]. Both methods for introducing an internal SECIS element have been shown to work but are not conducive to a general method of selenoprotein production given the required alteration of the protein coding sequence.

**Figure 3:**
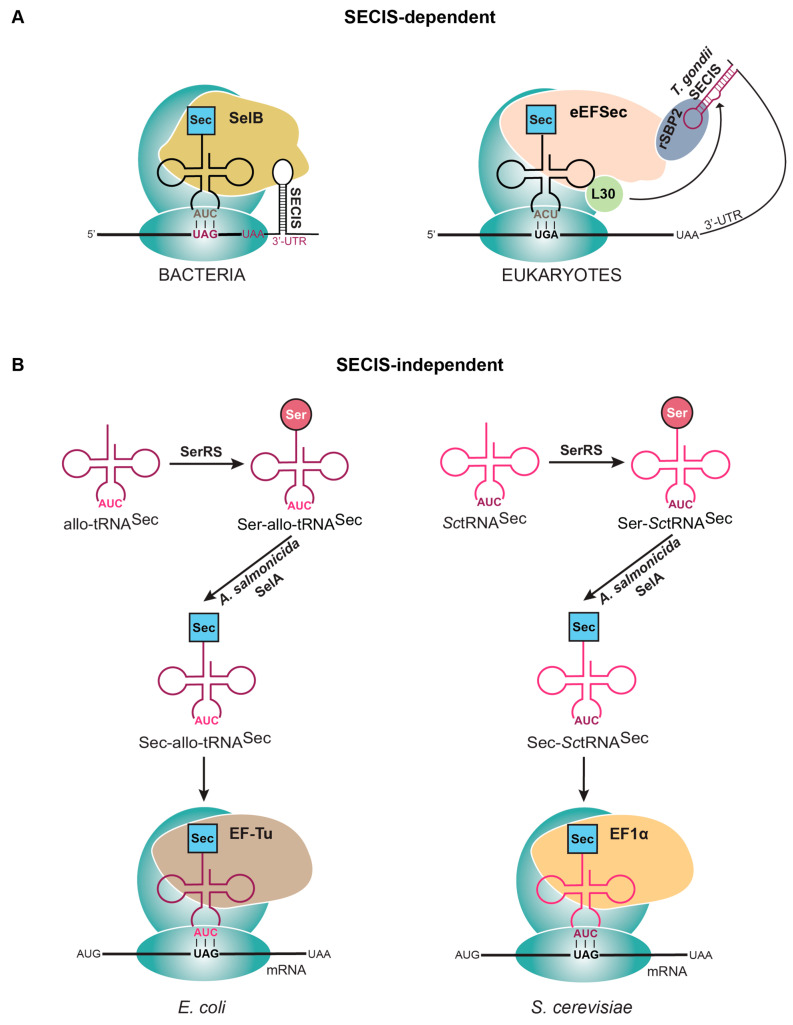
Strategies to express selenoproteins that (**A**) utilize the SECIS element or (**B**) are SECIS independent. (**A**) In bacteria, when the Sec to be inserted is near the C-terminal end of the protein, a SECIS element is placed right after the stop codon (UAA) in the 3′UTR. In eukaryotes, a SECIS element from *Toxoplasma gondii* is inserted downstream of the designated stop codon in the 3′UTR. (**B**) Removing the SECIS element in *Escherichia coli* and *Saccharomyces cerevisiae* requires the use of an engineered tRNA (allo-tRNA or *Sc*tRNA^Sec^) that is recognized by *Aeromonas salmonicida* SelA for conversion of Ser to Sec and the endogenous elongation factor (EF-Tu or EF-1α).

Alleviating this dilemma is possible with selenoproteins that contain Sec in the penultimate or ultimate position of the protein. This allows for a SECIS element to be engineered in the 3′-UTR of the bacterial mRNA, while still within the required parameters for SelB recognition. Since the SECIS element from each domain of life has a distinct structure [9], it is important to engineer a bacterial-like SECIS element into the 3′-UTR to promote SelB binding (Figure 3A). This was first performed using the eukaryotic enzyme thioredoxin reductase (TXNRD), in which a minimal SECIS from the *E. coli* selenoprotein formate dehydrogenase (FDH_H_) was placed after the stop codon. TXNRD has Sec in the penultimate position of the protein, providing the necessary proximity for SelB to recognize the SECIS element and efficiently produce active Sec-containing TXNRD [47]. Unlike standard recombinant protein expression, wherein the machinery for translation of the 20 canonical amino acids is abundant, the Sec-insertion machinery levels in a cell are much lower. This is because they are responsible for expressing much fewer proteins (3 in *E. coli* and 25 in humans) than the thousands of proteins encoded in the genome [48,49]. Therefore, in addition to strategically engineering the plasmid, it is necessary for bacteria to overexpress *selABC* genes (producing SelA, SelB, and tRNA^Sec^, respectively) to compensate for the increased selenoprotein mRNAs to be translated [50]. This strategy has been successfully used to produce a variety of selenoproteins [47,48].

A novel idea was inspired by the notion that using the SECIS in the aforementioned methods is limited to specific proteins without broad applicability. Therefore, with the knowledge of a minimal SECIS element capable of directing Sec insertion, the orientation of the SECIS element with SelB, and the ribosome structurally characterized [36], it was postulated that the SECIS element could be attached to the ribosome instead of on the mRNA. Having this design in mind, an orthogonal ribosome was engineered (RiboU) with a SECIS element in the 16S rRNA to facilitate Sec insertion at a UAG codon, using the natural Sec machinery (SelB, SelA, and tRNA^Sec^). To ensure that RiboU only recoded the desired transcript with Sec at UAG, the Shine–Dalgarno (SD) sequence of the mRNA was engineered to match the anti-SD sequence of RiboU [51]. This is the first evidence of unbiased selenoprotein production harnessing the natural Sec machinery. Further optimization and mechanistic understanding of this system will make this a useful tool for selenoprotein expression.

#### 4.1.2. Eukaryotes

In eukaryotes, EFSec has a similar role to SelB in bacteria, except that it does not bind to the SECIS element independently. Instead, SBP2 recognizes the SECIS element and bends it back towards EFSec [12]. As a result, the SECIS element does not have to be in such close proximity to the elongation factor, and is instead found up to 1600 nts away from the UGA codon in the 3′-UTR [52]. These requirements provide a framework more conducive to generating a plasmid for heterologous expression. On the other hand, though, there are additional protein factors involved in Sec translation that are not found in bacterial Sec insertion. Simply overexpressing all of these proteins may not be favorable for the cell to increase production of selenoproteins [53].

To develop a selenoprotein expression system in eukaryotes, the focus initially went to finding an appropriate SECIS element for this task. Eukaryotic SECIS elements have low sequence conservation, but the secondary structure is highly conserved and differs from what is observed in bacteria and archaea [9,53]. There is a characteristic segment of four non-Watson–Crick base pairs (termed the quartet) which is part of a kink-turn motif bound by SBP2 [12,53]. The remainder of the SECIS element sequence is not conserved and therefore it is not clear what sequence is required for efficient selenoprotein expression. Recent structural data show that the apical loop of the SECIS element is recognized by EFSec [12], but we still are not clear on the optimal sequence in this region for efficient translation. Through bioinformatic analyses, a non-canonical SECIS element was revealed in *Toxoplasma gondii* and verified to be highly active in the expression of SELENOH in mammalian cells. This information was harnessed to build a plasmid system (pSelExpress1) that fused this novel SECIS element to the 3′-UTR of the gene of interest (Figure 3A). Moreover, an additional copy of SBP2 was encoded under the EF-1α promoter to support the additional selenoprotein mRNA copies for expression [53]. This strategy has been successfully used for the expression of multiple eukaryotic selenoproteins [4].

Regulation of the selenoprotein expression by selenium concentration is another aspect for consideration that is not fully understood [54]. In selenium deficient conditions, it would make sense that the selenoprotein expression is downregulated. This is because in the absence of selenium the resulting translation product would not be accurate due to truncation or possible nonsense suppression. When selenium is low, certain selenoprotein mRNAs are more likely to be degraded through the nonsense-mediated decay (NMD) pathway. Since selenoproteins require Sec for their synthesis at a UGA codon these mRNAs are particularly susceptible to NMD should they not read through the stop codon [55]. There has been non-substantial observation when it comes to an overabundance of selenium. This paints quite a complex picture with regards to understanding the regulation of selenoproteins [54,56]. Though beyond the scope of this review, this is mentioned due to the notion that when expressing selenoproteins in eukaryotes, an appropriate level of selenium should be supplied (50–100 nM sodium selenite) [56].

### 4.2. Removing the SECIS Element

The SECIS element is critical to direct Sec insertion to the correct UGA codons in nature. However, when it comes to recombinant selenoprotein expression, it limits the possibility of site-specific Sec insertion. Here we discuss strategies that have been developed to overcome the requirement for having a SECIS in the mRNA to make selenoproteins.

#### 4.2.1. Harnessing the Natural System

In bacteria, the SECIS element is an important element for recognition by SelB. However, in the correct environment, it has been shown not to be essential for Sec insertion. Instead, the absence of a SECIS lowers the efficiency of the reaction. Therefore, the removal of competing factors (i.e., release factors), prevents termination and facilitates the insertion of Sec at the stop codon. This has been shown to be possible to insert Sec at a UAG codon in any position of a protein sequence provided the protein is expressed in recoded *E. coli* strains with release factor 1 removed (e.g., C321.ΔA) [57]. Given that the efficiency of the reaction is low, this strategy is susceptible to non-cognate suppression, often ending up with glutamine insertion at UAG [4].

#### 4.2.2. Engineering a Synthetic System

Due to the complexity of the natural Sec elongation system, another strategy sought to employ the elongation factor used for the other 20 canonical amino acids (EF-Tu in bacteria and EF-1α in eukaryotes). This required engineering tRNAs that could facilitate Sec synthesis and be introduced into the ribosome via EF-Tu (or EF-1α). Initial efforts to create a SECIS-independent system in bacteria made a hybrid tRNA composed of parts from both *E. coli* tRNA^Ser^ and tRNA^Sec^ (tRNA^UTu^, tRNA^SecUx^, tRNA^UTuX^, tRNA^UTuT^ variants) [4]. Further optimization found that instead of adapting an endogenous tRNA, introducing a tRNA not found in *E. coli* was more efficient. These tRNAs (allo-tRNAs) were found from metagenomic datasets and contained a 12 bp acceptor domain (in a 9/3 orientation) that is recognized by EF-Tu, a D-arm recognized by SelA (6 bp stem and 4 base loop), and a long variable arm for initial serylation by SerRS [58]. These characteristics made them perfect candidates for a SECIS-independent translation system that employed EF-Tu for elongation (Figure 3B). Further studies went on to develop a plasmid system for site-specific Sec insertion the contained the following: gene for an allo-tRNA, engineered *Aeromonas salmonicida* SelA, *A. salmonicida* SelD, *Treponema denticola* thioredoxin, and *E. coli* cysteine desulfurase (SufS) (pSecUAG-Evol2, Addgene #163148) [42,58].

More recently, a similar strategy was followed to develop Sec-insertion technology in *Saccharomyces cerevisiae*, an organism that does not have its own mechanism to insert Sec [59]. In the same way as described in bacteria, the main focus for a SECIS-independent translation system was through tRNA engineering. The tRNA design for *S. cerevisiae* was inspired by the bacterial system, using the major *S. cerevisiae* tRNA^Ser^ isoacceptor as a scaffold and making changes to represent *A. salmonicida* tRNA^Sec^. It followed that *A. salmonicida* SelA and SelD [58] were also required in *S. cerevisiae* to facilitate Sec insertion (Figure 3B). One additional requirement, selenocysteine lyase, was included to promote the turnover of selenocysteine. This gene is naturally present in *E. coli*, because of their capability to express selenoproteins [60], but is absent in yeast. Therefore, selenocysteine lyase from *Mus musculus* was expressed and found to improve growth of the cells and promote Sec insertion [59].

## 5. Expression of Selenoproteins In Vitro

Given the limitations of cellular protein expression and competition between termination of protein synthesis and Sec insertion, techniques have emerged to avoid this. These techniques include using cell-free systems and chemical synthesis methods.

### 5.1. Cell-Free Systems

#### 5.1.1. Bacterial Cell-Free Systems

In vitro protein expression provides the advantage of easily removing or adding components to the translation reaction. This provides a platform for optimizing translation reactions and controlling their products. However, it does not abrogate the requirement of using the complex translation path required for selenoprotein production, and therefore similar stipulations are present, as discussed for in vivo approaches. In a bacterial-based system, the mRNA requires a SECIS element for recognition by SelB. Using this strategy, *E. coli* FDH_H_ was expressed with a PURExpress kit (New England Biolabs Inc., Ipswich, MA, USA) supplemented with Sec-tRNA^Sec^ and SelB. FDH_H_ is a natural selenoprotein in *E. coli*; therefore, cloning the native mRNA sequence into a bacterial expression vector provided the coding information and SECIS element required for in vitro selenoprotein expression [27].

On the other hand, to express unnatural bacterial selenoproteins, the cell-free system must also rely on previous tRNA engineering for a SECIS-independent, EF-Tu reliant path. Work conducted in this area has leaned towards recoding UAG with Sec which means that RF1, responsible for terminating translation at UAG codons, can be removed from the reaction to promote Sec insertion. This was performed using the PURExpress ΔRF123 kit (New England Biolabs Inc., Ipswich, MA, USA) in the absence of RF1 and supplemented with Sec-tRNA^Sec^ and EF-Tu [27]. In both approaches, there is a requirement for tRNA^Sec^ to be charged with Sec. Due to the requirement of Sec to be biosynthesized on the tRNA, this is not trivial, as was discussed above.

**Figure 4:**
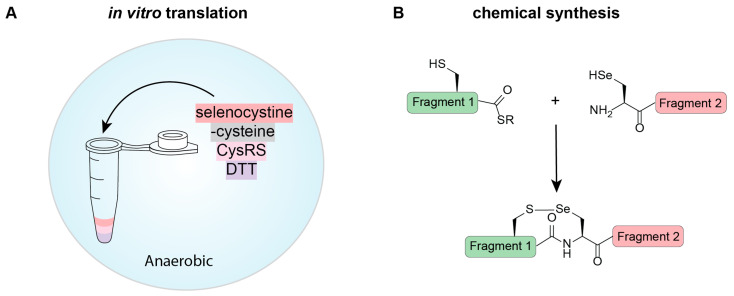
Cell-free methods for selenoprotein expression involving (**A**) in vitro protein expression and (**B**) chemical synthesis. (**A**) Selenoproteins can be expressed using in vitro translation systems based on the previously described in vivo methods. Here we show one method that cannot be replicated in vivo, which involves acylating tRNA^Cys^ with Sec using CysRS. Under the correct reducing conditions (DTT in an anaerobic environment), selenocystine is reduced to Sec and can be used as a substrate for aminoacylation. In the absence of Cys, all codons encoding for Cys will contain Sec. (**B**) Native chemical ligation involves the fusion of two peptide fragments together. These can be expressed in a host or chemically synthesized. For selenoproteins, Sec is an active amino acid in the ligation reaction and, with chemical synthesis into a peptide, can facilitate the formation of a selenoprotein.

An alternate method to the use of an engineered tRNA capable of Sec biosynthesis and EF-Tu recognition, has been to mischarge tRNA^Cys^ with Sec. This is an approach that is possible in proteins with no Cys residues, or where all Cys residues permit Sec substitution. In a cell-free system, Cys is removed from the reaction and replaced with selenocystine (the oxidized form of Sec). In a strategically determined reduced environment (10 mM dithiothreitol [DTT]), the diselenides are broken apart, providing available Sec for CysRS to misacylate tRNA^Cys^ with (Figure 4A). Performing this reaction in an anaerobic environment provides sufficient DTT to reduce the diselenides without reducing selenocysteine further to elemental selenium [61].

#### 5.1.2. Eukaryotic Cell-Free Systems

Development of an in vitro translation system requires knowledge and reconstitution of all the necessary proteins and factors. When it comes to eukaryotic selenoprotein production, though, the details are not as clear and understood as bacteria. It is agreed from in vitro studies and structural analysis that the core ingredients for in vitro selenoprotein expression include EFSec, Sec-tRNA^Sec^, and SBP2 [12,39]. Low levels of Sec insertion, however, raise the question of whether there are additional factors that improve this process. The ribosomal protein, L30, has been shown in vitro to compete with SBP2 for binding to the SECIS element [62,63], but it has not been tested for its essentiality in selenoprotein translation [39]. Other components which have yet to be tested for optimal in vitro expression, include nucleolin, SECp43, GAPSec, and eIF4a3 [39,64].

### 5.2. Chemical Synthesis and Ligation

#### 5.2.1. Native Chemical Ligation

Synthesizing peptides chemically is becoming a more affordable technique; and therefore, in some proteins, is a viable option for production. This is especially relevant when trying to make difficult-to-express proteins, or going beyond the canonical amino acids, such as in selenoproteins. Native chemical ligation (NCL) technology provides the opportunity to prepare proteins up to ~200 amino acids in length [65] with strategic placement of ligation sites. NCL originally relied on the presence of an N-terminal Cys for ligation; however, with the similarity in chemical properties between Cys and Sec, the use of Sec for NCL is possible (Figure 4B) [1]. Importantly, the unique chemical features of Sec cause NCL reactions to proceed 1000-fold faster than Cys reactions, opening the possibility for chemoselective alkylation of Sec residues. While there are often many Cys residues in a protein to facilitate multiple ligations, there is often only a single Sec in selenoproteins for this purpose. This is not discouraging because Sec can be deselenized to alanine under anaerobic conditions. This characterized chemical reaction widens the possible positions that can be used for ligation and expands the protein repertoire that can benefit from chemical synthesis.

With that said, the strategy for multiple ligations with Sec requires careful consideration and planning. Standard stepwise Fmoc-solid-phase peptide synthesis is used to prepare the peptides for ligation; however, due to the oxidation properties of Sec, it is not a possible substrate. This has since been overcome by using selenazolidine (Sez), a protected form of Sec, as a substrate for synthesis of the peptides for ligation. Once synthesized, the Sec residue can deprotected, following treatment by methoxyamine hydrochloride, and is ready for ligation. If only a single ligation is required, then the resulting protein would have Sec at the desired position. Otherwise, when multiple ligations are necessary, the undesired Sec residues are converted to alanine through an anaerobic deselenization reaction with Tris (2-carboxyethyl) phosphine (TCEP) in the presence of DTT. Sodium ascorbate is also added in these reactions to inhibit undesired deselenization (or desulfurization) reactions. SELENOM has been successfully prepared in this manner, involving three Sez-driven NCL ligations, two deselenizations, and a methoxyamine hydrochloride reaction [65].

#### 5.2.2. Expressed Protein Ligation

Most human selenoproteins have a single Sec residue, while the remainder of the protein is composed of canonical amino acids. As a result, the protein sequence containing the canonical amino acids can be recombinantly expressed in *E. coli*, leaving only the Sec-containing part of the protein to be chemically synthesized. Conveniently, often the Sec is near the end of the selenoprotein, reducing the need for chemical synthesis of large Sec-containing peptides. With this in mind, much of the strategic planning for multiple ligations can be avoided with the focus only on the Sec-containing region [4].

## 6. Discussion and Conclusions

Selenoproteins are crucial for human health, with many of them playing roles in oxidative stress to maintain proper cellular function [2,3,5]. Understanding the detailed functional mechanism of selenoproteins and how Sec is incorporated into them is vital for an advancement in selenoprotein research. However, there are three major stumbling blocks that have impeded exponential growth in this research area. These include the following: (i) the instability of Sec, (ii) the complicated aminoacylation process for Sec onto its tRNA, and (iii) the multi-step restrictive mechanism for selenoprotein expression. To overcome these three barriers, novel strategies have been implemented and developed to study selenoprotein synthesis. With the knowledge that aminoacylation of Sec involves a Ser intermediate [14,15], which is a much easier amino acid to work with, researchers have been able to understand selenoprotein synthesis further, with Ser or even with no amino acid at all. However, this is sometimes a tricky question to ask. Therefore, through this review, we have collected and discussed when it is appropriate to work with an alternate amino acid to Sec, and what information can be gained with this method. Moreover, we have highlighted the strategies and breakthroughs that have stimulated progress in the selenoprotein field.

Investigation into the process of selenocysteinylation requires the correct amino acid to be used [20]. Because Sec is labile, the addition of SelD is required in reaction mixes to convert sodium selenite (commercially available) into the selenophosphate precursor to complete the conversion of Ser to Sec. Studying bacterial selenocysteinylation is easier than archaeal or eukaryotic, because there is only one intermediate (Ser-tRNA^Sec^). The additional step of phosphorylation (to obtain Sep) before conversion to Sec in archaea and eurkayotes, adds an extra intermediate [21,22,23]. Distinguishing the affinity of each enzyme in these reactions involves purification of the intermediate states. This laborious process is often avoided, and instead ratios between the intermediate amino acids are used to investigate tRNA identity elements for each enzyme, furthering our understanding of the aminoacylation path.

After tRNA^Sec^ is aminoacylated with Sec, the next step involves translation through the ribosome. Unique to standard amino acids, Sec-tRNA^Sec^ requires additional steps and factors, such as a specialized elongation factor and the SECIS element, at minimum [12,52]. Bacterial translation systems have been well-studied; while in archaea and eukaryotes, a complete picture is still being assembled. This is due to the additional factors required in these more complex organisms. Kinetic studies of Sec elongation factors reveal different affinities for Ser-tRNA^Sec^ compared to Sec-tRNA^Sec^, suggesting that when studying translation, Sec is the preferred amino acid. This has proven to be true in bacteria; however, recent data in eukaryotes have found that the Sec-specific elongation factor (EFSec) can recognize and translate Ser-tRNA^Sec^ in vitro (Figure 3B). This trick has alleviated the requirement of Sec for studying the translation complex in eukaryotes but infers that caution should be taken with the interpretation of the results.

In addition to understanding the natural translation mechanisms for Sec insertion, immense research has been dedicated to achieving a robust platform for recombinant selenoprotein expression. To identify the role that Sec has in selenoproteins, there is a need to compare a Sec-containing protein with a Cys-containing protein. Strategies for selenoprotein expression have been engineered in bacteria, in eukaryotes, and in vitro. In bacteria, three methods have adopted the natural Sec machinery for this purpose, to install a minimal SECIS element as follows: (i) in the translated region of the mRNA, (ii) in the 3′-UTR of the mRNA, and (iii) in the rRNA. Other strategies have strayed away from the natural Sec system and focused on using the canonical machinery for amino acid incorporation, namely bacterial EF-Tu. Development of this latter mechanism now permits the use of studying translation mechanisms with Ser instead of Sec. This is because EF-Tu does not have the same preference for amino acid that SelB has; and, rather, just requires the presence of an amino acid [66]. With that said, crystal structures have been solved with tRNA^Sec^ and the Sec-specific elongation factor (and other enzymes), in the absence of an amino acid [11,12]. This is possible because the process of crystallization requires a high concentration of materials (higher than the affinity of the reaction), which can induce binding of tRNA to the protein without the need for it to be aminoacylated. Recombinant selenoprotein expression has also been developed in *S. cerevisiae*, an organism that does not naturally have this machinery [59].

There are limitations in cellular expression; thus, prompting the expansion of selenoprotein expression research in vitro through cell-free systems and chemical ligation (Figure 4) [65]. Bacteria have a cell-free system that has been shown to be successful, while eukaryotic cell-free systems still require optimization. However, to avoid of the need for specialized elongation factors, tRNAs, and SECIS elements, chemical ligation has been employed to harness the chemical reactivity of Sec. Chemical ligation uses a protected form of Sec (Sez), and with careful planning can involve multiple ligations for production of selenoproteins [65]. This method has provided an alternative to the cell-based strategies developed and opens the possibilities to use Sec in proteins that contain non-canonical amino acids (Figure 4B).

In conclusion, selenoproteins play a critical role in maintaining human health and preventing a wide range of diseases. Despite the challenges in studying and synthesizing these proteins, significant progress has been made through innovative approaches and advanced techniques. Ongoing research is essential to fully understand the mechanisms of Sec incorporation and to develop efficient methods for producing human selenoproteins. This knowledge not only furthers our understanding of cellular processes but holds promise for developing therapeutic strategies to combat diseases related to selenoprotein deficiencies. As the field advances, the potential for a breakthrough in medical and biotechnological applications continues to grow, underscoring the importance of the continued investment and exploration in selenoprotein research.

## Data Availability

The original contributions presented in the study are included in the article; further inquiries can be directed to the corresponding author/s.
